# Predictive Value of Serum Antibodies and Point Mutations of AQP4, AQP1 and MOG in A Cohort of Spanish Patients with Neuromyelitis Optica Spectrum Disorders

**DOI:** 10.3390/ijms20225810

**Published:** 2019-11-19

**Authors:** Pablo García-Miranda, Francisco J. Morón-Civanto, Maria del Mar Martínez-Olivo, Nela Suárez-Luna, Reposo Ramírez-Lorca, Lucía Lebrato-Hernández, Raquel Lamas-Pérez, Guillermo Navarro, Javier Abril-Jaramillo, Maria Isabel García-Sánchez, José Luis Casado-Chocán, Antonio José Uclés-Sánchez, Mercedes Romera, Miriam Echevarría, María Díaz-Sánchez

**Affiliations:** 1 Instituto de Biomedicina de Sevilla (IBiS), Hospital Universitario Virgen del Rocío/CSIC/Universidad de Sevilla, 41013 Seville, Spain; pgarcia2@us.es (P.G.-M.); mcivanto-ibis@us.es (F.J.M.-C.); marmaroli1@alum.us.es (M.d.M.M.-O.); mnsluna@gmail.com (N.S.-L.); reporamirez@us.es (R.R.-L.); 2 Unidad de Gestión Clínica de Neurociencias, Servicio de Neurología del Hospital Universitario Virgen del Rocío, 41013 Sevilla, Spain; lucia.lebrato.hdez@gmail.com (L.L.-H.); rlamas90@hotmail.com (R.L.-P.); jcasadoch@gmail.com (J.L.C.-C.); antonioj.ucles.sspa@juntadeandalucia.es (A.J.U.-S.); 3 Servicio de Neurología del Hospital Universitario Virgen Macarena, 41009 Sevilla, Spain; guillermo.navarro.sspa@juntadeandalucia.es (G.N.); javierabriljaramillo@hotmail.com (J.A.-J.); maribel.garsan@gmail.com (M.I.G.-S.); 4 Servicio de Neurología del Hospital Universitario Virgen de Valme, 41014 Sevilla, Spain; romera.neu@gmail.com

**Keywords:** demyelinating disease, NMOsd, AQPs, MOG, gene sequencing, immunohistochemistry

## Abstract

The detection of IgG aquaporin-4 antibodies in the serum of patients with Neuromyelitis optica (NMO) has dramatically improved the diagnosis of this disease and its distinction from multiple sclerosis. Recently, a group of patients have been described who have an NMO spectrum disorder (NMOsd) and who are seronegative for AQP4 antibodies but positive for IgG aquaporin-1 (AQP1) or myelin oligodendrocyte glycoprotein (MOG) antibodies. The purpose of this study was to determine whether AQP1 and MOG could be considered new biomarkers of this disease; and if point mutations in the gDNA of *AQP4*, *AQP1* and *MOG* genes could be associated with the etiology of NMOsd. We evaluated the diagnostic capability of ELISA and cell-based assays (CBA), and analyzed their reliability, specificity, and sensitivity in detecting antibodies against these three proteins. The results showed that both assays can recognize these antigen proteins under appropriate conditions, but only anti-AQP4 antibodies, and not AQP1 or MOG, appears to be a clear biomarker for NMOsd. CBA is the best method for detecting these antibodies; and serum levels of AQP4 antibodies do not correlate with the progression of this disease. So far, the sequencing analysis has not revealed a genetic basis for the etiology of NMOsd, but a more extensive analysis is required before definitive conclusions can be drawn.

## 1. Introduction

Optic neuromyelitis (NMO) is an autoimmune, inflammatory and demyelinating disease of the central nervous system (CNS) which mainly affects the optic nerve and the spinal cord. Patients with this disease usually suffer recurrent and severe episodes of optic neuritis and transverse myelitis that lead to severe unilateral or bilateral vision loss, and motor and sensory compromise below the medullary level of the lesion that often leads to loss of sphincter control [1]. Although the associated mortality is not very high, its clinical course is usually characterized by progressively more debilitating outbreaks leading to irreversible and severe neurological deficits from the initial phases of the disease (frequently the patients develop blindness and/or hemiplegia/paraplegia within 5 years after disease onset). Therefore, early diagnosis is crucial for establishing a disease-modifying treatment from its early stages and thus preventing the incapacitating progression of this disease [1]. The development of diagnostic tests with high sensitivity and specificity is greatly needed, and its etiology remains poorly understood. Today it is classified as a rare disease. Although for a long time it was considered a subtype of multiple sclerosis (MS), new radiological, serological, and pathological findings have clearly defined NMO as a distinct entity from MS [1]. The main evidence for such a distinction was provided by Lennon et al. in 2004, who discovered the presence of specific and sensitive immunoglobulins (IgG-NMO) in the serum of patients with NMO and but not in the classic forms of MS [2]. The antigen recognized by the IgG-NMO was found to be Aquaporin-4 (AQP4), the water channel with the highest expression in the CNS [1,2,3,4]. In recent years, different studies have shown that the presence of anti-AQP4 antibodies in patients is highly predictive of NMO [5]. The most recent revision of the diagnostic criteria for this pathology, published in 2015, has broadened the scope of NMO to what is now known as NMO spectrum disorders (NMOsd) [6]. NMOsd includes limited forms of the disease (optic neuritis without myelitis and transverse myelitis without optic neuritis) and other less frequent clinical conditions whose lesions settle in areas of the CNS with high expression of AQP4 [6,7].

Unfortunately, just over 20% of the patients included in the NMOsd group are seronegative for the anti-AQP4 antibody [5,6,8]. We know that in addition to AQP4, human astrocytes of the CNS also express Aquaporin-1 (AQP1), and specifically there has been very high AQP1 expression in areas prone to develop NMO lesions, such as the spinal cord, optic nerves and cerebral white matter [9]. Functional role of AQP1 in these locations has been associated to its water permeability capacity. In this sense some authors [10,11,12] have indicated that a subgroup of patients with suspected NMOsd, seronegative for anti-AQP4-IgG, present anti-AQP1 antibodies in serum, which could mean a new classification biomarker for these demyelinating CNS disorders. Continuing the search for potential NMOsd biomarkers, especially in cases with a high clinical suspicion but seronegative for anti-AQP4 antibodies, independent groups have repeatedly identified the presence of antibodies against oligodendrocyte myelin glycoprotein (anti-MOG) in the serum of 7–20% of adult patients with NMOsd, which represents 20–35% of seronegative cases for anti-AQP4-IgG [13]. MOG is a glycoprotein of the immunoglobulin superfamily that, although it represents a minority component of myelin (0.05%), is of great importance as a surface marker for the maturation of oligodendrocytes. The MOG protein and the antibodies directed against it have been studied for years, although its pathogenic role in the demyelinating diseases of the CNS is still unknown (see [14] for review). This antibody has been described as being associated with different acquired demyelinating diseases in both pediatric and adult patients, although the frequency varies depending on the clinical syndrome and the age of the patients. Acute disseminated encephalomyelitis (ADEM) is the clinical form that is most frequently associated with anti-MOG (40%), but these antibodies are also detected in NMOsd [15].

Although this is an interesting finding, it is still necessary to confirm the presence of antibodies against AQP1 and MOG in additional series of patients. It would also be useful to know whether the presence of these antibodies is related to a specific NMOsd phenotype.

On the other hand, the observation of familial aggregation of NMOsd suggests that genetic factors may play a role in the pathogenesis of this disease, as is the case with other autoimmune neurological disorders, such as MS [16]. Among the genetic factors, some studies have revealed an association of the major histocompatibility complex (MHC) region with NMOsd [17]. In addition, the *AQP4* gene has been analyzed in several populations, mainly in Asian NMOsd patients, since the pathogenic contribution of anti-AQP4 antibodies to the development of these disorders, at least in experimental animal models, is now well-accepted [18]. However, regarding the association between variations in the *AQP4* gene and NMOsd [19,20,21,22] conflicting results have been reported so far, and there are no studies available to date concerning the association between the *MOG* and *AQP1* genes and NMOsd.

In this study we examined the presence of serum IgGs against AQP4, AQP1 and MOG using two cell-based assay (CBA) methods, one previously developed by us [23,24] and the commercial Euroimmun^®^ Assay widely used abroad for diagnostic purposes. ELISA determinations for the three antibodies were also performed by either commercial, as for AQP4 and MOG, or by in-house developed methods in the case of AQP1-IgG detection. Furthermore, we conducted a preliminary genetic analysis of these three candidate genes, to investigate whether there are genetic variants associated with NMOsd (anti-AQP4-IgG-positive and anti-AQP4-IgG-negative) in Spanish patients. For this, we performed a direct DNA-sequencing analysis of all exons encoding these genes in a small cohort of patients with NMOsd; and additionally, we investigated the presence of new genetic variants of the three genes in another small cohort of patients with MS, finding that the genetic susceptibility for NMOsd is markedly different from that of MS patients. In summary, our results confirm the high sensitivity and specificity of CBA for detecting antibodies against AQP4, only present in NMOsd cases. Neither anti-MOG-IgG, nor anti-AQP1-IgG were detected in NMOsd patients. The genetic analysis we performed here indicates that there are different variations of the *AQP1*, *AQP4*, and *MOG* genes in patients with NMOsd, which are neither necessary nor sufficient for developing NMOsd. However, a comprehensive analysis of the *AQP4* gene in a large independent population would be necessary to derive more definitive conclusions. Further experiments by other groups would be necessary to strengthen and support the findings presented in this study.

## 2. Results

### 2.1. Determination of Antibodies against AQP4, MOG, and AQP1

We addressed two aspects of NMOsd, one related to the methods for diagnosis and the other associated with the etiology of this disease. The study included 119 subjects (81 female) classified into 7 groups based on their medical diagnosis, as shown in Table 1. The presence of serum antibodies against AQP4, MOG, and AQP1 was determined by using CBA and ELISA detection systems either developed in our own laboratory or using commercially available kits. A summary of these results, together with the demographic and clinical characteristics of the patients is presented in Table 1. First, we began by comparing the results obtained with the CBA we developed some years ago [23] for detecting anti-AQP4 antibodies in serum, with the results obtained using the commercially-available CBA from Euroimmun. Our results demonstrate that anti-AQP4 antibodies were only detected in the serum of NMOsd patients and were never detected in the serum of patients from any other disease group analyzed or in the control subjects. Specifically, similar detection sensitivity was obtained with the commercial CBA as with the in-house developed [23] detection system. The serum of twelve out of the 18 (67%) NMOsd patients was positive for these antibodies using the commercial CBA, compared with 11 (61%) who tested positive using the protocol developed in our laboratory [23]. A much lower sensitivity for detection of AQP4 antibodies was obtained with the commercial ELISA kit, with which the serum of only 7 (39%) patients tested positive.

MOG antibodies were only detected in the serum of 1 patient who was diagnosed with recurrent idiopathic myelitis (RMy, Table 1), and only when using the commercial CBA system, not the commercial ELISA system. Finally, anti-AQP1 antibodies were not detected in any of the patients, with either the CBA or ELISA, although control experiments showed that both detection assays were functioning properly, as shown below.

### 2.2. Comparison of CBA Results for Anti-AQP4 Antibody Determinations

**Figure 1:**
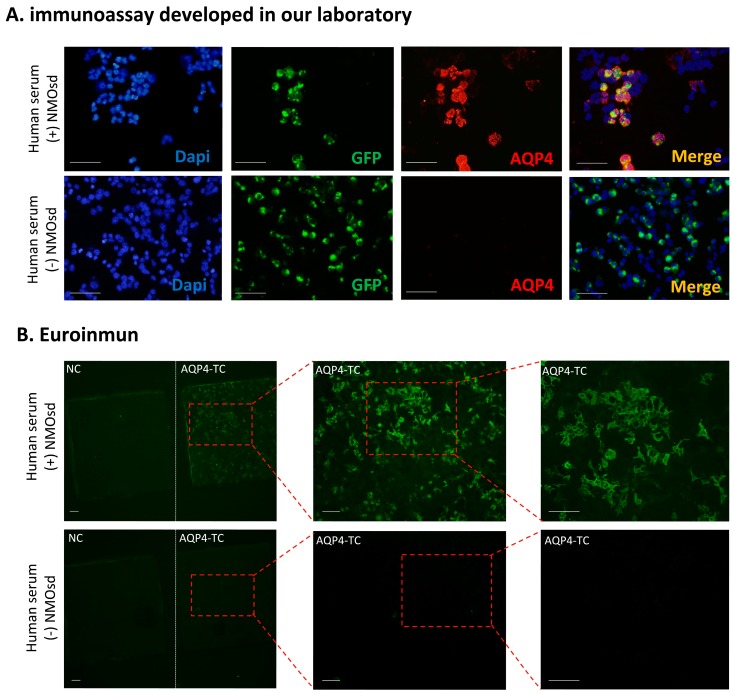
Immunofluorescence assay for the detection of anti-aquaporin-4 (AQP4) antibodies: (**A**), representative images of the immunofluorescence assay developed in our laboratory using cells expressing human AQP4 fused to green fluorescent protein (GFP) (hAQP4-EGFP) (green). The immune reaction produced by patient serums either from Neuromyelitis optica spectrum disorder (+NMOsd) or not (−NMOsd) over AQP4 expressing cells (red) revealed presence or absence of anti-AQP4 antibodies in analyzed serums. Nuclei were visualized with Dapi (blue) and the merged image of both fluorescent signals (anti-AQP4 antibodies and GFP) is shown in yellow. (**B**), representative images of the Euroimmun kit using patient serums either from Neuromyelitis optica spectrum disorder (+NMOsd) or not (−NMOsd) over either AQP4 expressing cells (AQP4-TC) or not AQP4 expressing cells (NC) as negative control. The images in both cases (**A**,**B**) were obtained using the sera from the same patients (+NMOsd and –NMOsd). The immune reaction produced by NMOsd patient serums were visualized in green. Scale bar 100 µm.

The serum of all 119 patients was analyzed using both CBA methods, obtaining very similar results. A discrepant result was obtained in only one sample (1/119) that was diagnosed as positive for AQP4 antibodies by the commercial method, while appeared negative when analyzed by our own assay. Detection of anti-AQP4 antibodies in all the rest of serums (118/119) yielded matching results. Figure 1 shows images from representative examples of both immunofluorescent CBA methods. In our own assay, an overlapping signal from green fluorescent protein (GFP in green) and AQP4 protein (in red) leads to a merged signal (in yellow) confirming the presence of anti-AQP4 antibodies in human serum NMOsd(+). A merge signal was always absent in anti-AQP4 (–) serum. In the commercial assay, a negative control with cells untransfected for AQP4 is included in the biochip slide and allows for a side-by-side comparison of positive and negative results. A fluorescent signal was only present over AQP4-transfected cells when NMOsd(+) serum was examined. A fluorescent signal was never observed in the slide lot for untransfected cells, or when NMOsd(–) serum was analyzed.

### 2.3. Comparison of CBA and ELISA Results for Anti-AQP4 Antibody Determinations

The commercial kit used for the ELISA determination of anti-AQP4 IgG, showed high variability and less accuracy for identifying the presence of anti-AQP4 antibodies. The results obtained by ELISA were highly specific since the serum from NMOsd(–) patients was always negative for the assay (Table 1); but showed a lower sensitivity than the CBA assays, since only 7 patients tested positive out of the 18 NMOsd(+) patients; in contrast with the higher numbers of anti-AQP4-positive patients, 11 and12, confirmed by the two types of CBA analysis, our own method or the commercial kit, respectively (Table 1).

### 2.4. Analysis by CBA and ELISA of Anti-MOG Antibodies

**Figure 2:**
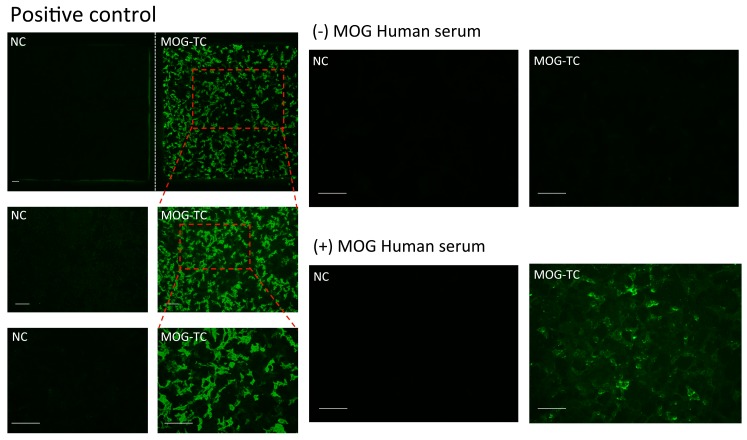
Immunofluorescence assay for the detection of anti-MOG antibodies. Representative images of the Euroimmun kit using patient serum and positive control serum over either MOG expressing cells (MOG-TC) or nor MOG expressing cells (NC) as negative control. The immune reaction produced by patient serums with anti-MOG antibodies were visualized in green. Scale bar 100 µm.

Two commercial assays were used for anti-MOG IgG determinations; the Euroimmun kit for CBA and the SensoLyte kit for ELISA. As can be seen in Table 1, only 1 out of the 119 patients analyzed resulted positive for the presence of MOG antibodies, and that patient had been diagnosed with recurrent idiopathic myelitis (RMy) and the positive result was obtained using the CBA system, as shown in Figure 2. No fluorescence was observed when MOG(–) serum was analyzed, or whenever non-transfected cells (negative control), were exposed to MOG(–) serum. In comparison, the results obtained using a positive control serum included in the kit produced a strong signal when MOG-transfected cells were exposed to the positive serum, whereas no fluorescent signal was observed for non-transfected cells. The results of the ELISA experiments revealed the absence, or undetectable levels, of anti-MOG antibodies in all the samples analyzed (Table 1), and were negative even for the patient who tested positive with the CBA, as shown in Figure 2.

### 2.5. Analysis by CBA and ELISA of Anti-AQP1 Antibodies

**Figure 3:**
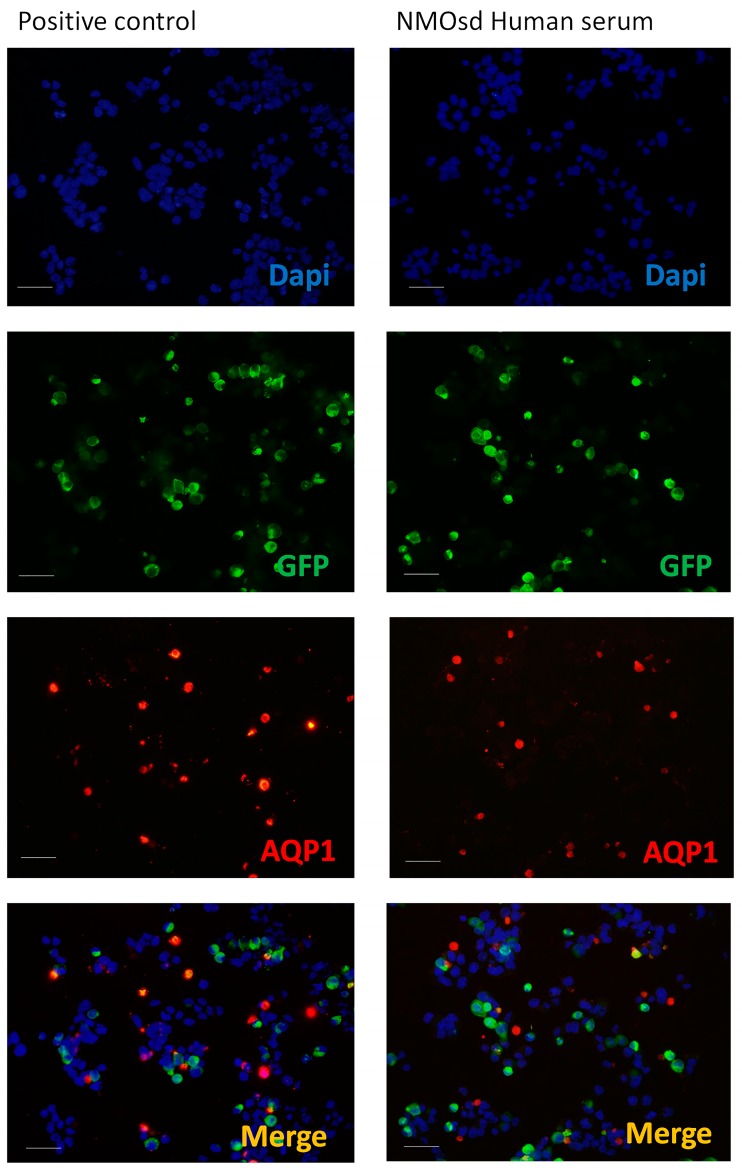
Immunofluorescence assay for the detection of anti-AQP1 antibodies. Representative images of the immunofluorescence assay developed in our laboratory using cells expressing human aquaporin-1 (AQP1) (hAQP1-EGFP) fused to GFP (green). The immune reaction produced by anti-AQP1 antibody (ab15080, ABCAM) (left panels) and by patient serums from Neuromyelitis optica spectrum disorder (+NMOsd) (right panels) over AQP1 expressing cells (red). Nuclei were visualized with Dapi (blue) and the merged image of both fluorescent signals (anti-AQP1 antibodies and GFP) is shown in yellow. Scale bar 100 µm.

A CBA method similar to the one we developed previously [23] for the detection of anti-AQP4 IgG was used here for the detection of anti-AQP1 IgG. Figure 3 shows the results from these immunofluorescent assays, presenting a side-by-side comparison of the results obtained when AQP1 transfected cells were exposed to a commercial antibody against AQP1 (positive control) or when serum from an NMOsd patient was used as the primary antibody. In this assay, cells transfected with AQP1 also expressed the fluorescent protein GFP. Therefore, when a commercial antibody that recognizes AQP1 was used, a double fluorescent signal, or a merge signal (yellow/orange), was obtained over those cells (positive control) expressing AQP1. By contrast, serum from an NMOsd patient did not show a double fluorescent signal in any cell (Figure 3), indicating that labeling observed over certain cells (red) is non-specific and does not correspond to the expression of AQP1. The serum of all 119 patients included in the study was examined by this CBA method and a merge signal (yellow cells) over AQP1 expressing cells was never observed, confirming the absence of anti-AQP1 IgG in the serum of all patients examined.

ELISA experiments using a commercial peptide for AQP1 fixed to the bottom of the plate revealed the total absence, or undetectable levels, of anti-AQP1 antibodies in all the samples analyzed (Table 1).

### 2.6. Time Course Progression of Anti-AQP4 IgG Levels in NMOsd(+) Patients

**Figure 4:**
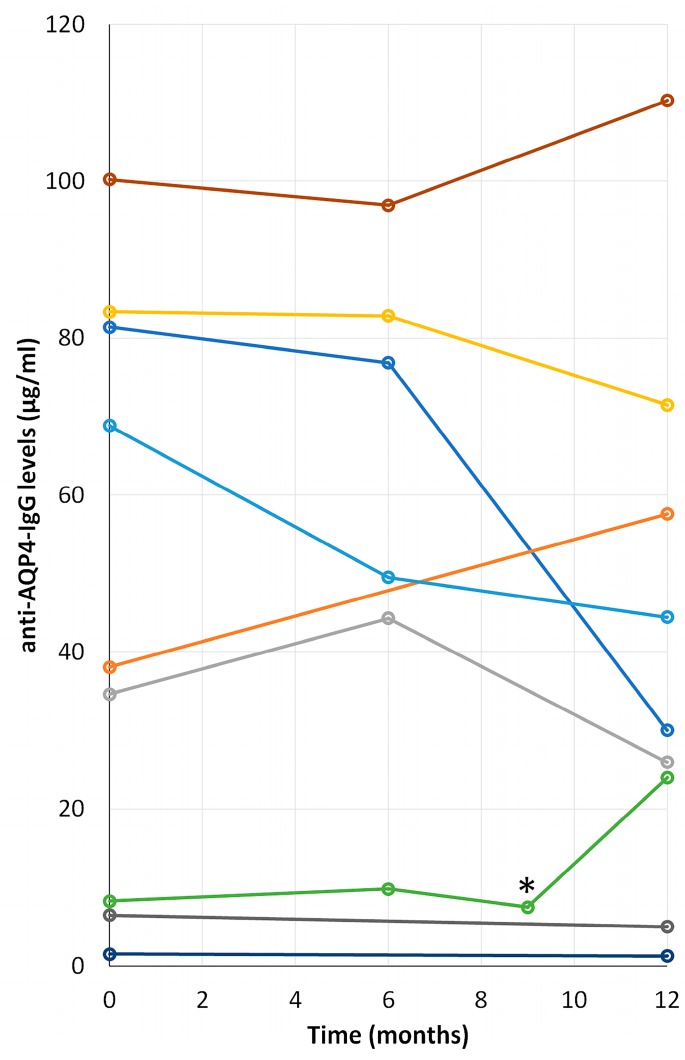
Time course progression of anti-AQP4-IgG levels in NMOsd(+) patients. Levels of IgG-anti-AQP4 in serum of 9 patients along a period of 12 months. *, sample obtained in a crisis or clinical episode.

A follow up of the levels of anti-AQP4 IgG in serum during a period of 12 months, which started with the first determination of these antibodies, was performed for 9 out of the 12 positive patients included in the study (Table 1). Mobility and displacement problems to come to the service for blood extraction, the lack of consent for inclusion in the analysis, and the death of one of the patients, were the reasons for the failure to include three patients in these analyses. At the time of the first determination of anti-AQP4 antibody all patients were already receiving an immunosuppressive treatment (azathioprine, mycophenolate mofetil or rituximab; and during the follow-up, no patient changed the drug or changed their dose. The results are plotted in Figure 4, which illustrates the large outcome variability obtained. In two cases, the levels remain pretty much constant over time; however, by the end of the period, levels of anti-AQP4 IgG increased in three patients and decreased in the other four. Disease relapses do not seem to affect the levels of serum anti-bodies, as was shown by the analysis performed on one of our patients, marked with an asterisk in Figure 4.

### 2.7. Genomic DNA Analysis of Variants in AQP4, AQP1 and MOG Genes

To screen for possible functional or causative variants in the *AQP4*, *AQP1* and *MOG* genes, we sequenced all exons of the three genes in 18 NMOsd Spanish patients (12 seropositive and 6 seronegative for anti-AQP4 IgG) and in 16 MS patients for comparative purposes.

**Figure 5:**
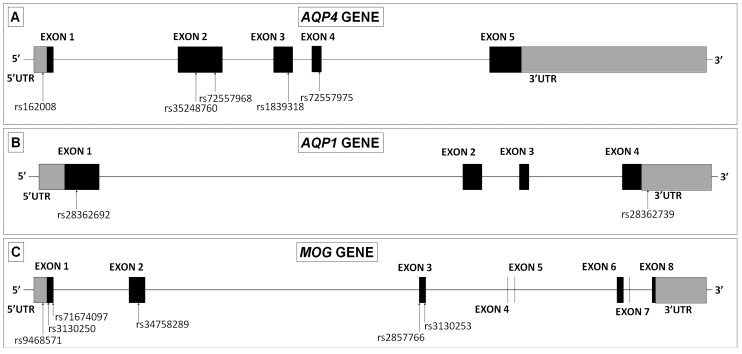
Squeme of exons organization in AQP4, AQP1 and MOG genes. Different variants found per each gene in NMOsd(+) patients.

The *AQP4* gene consists of five exons spanning a fragment of 13.7 Kb. We identified five single nucleotide DNA variants. The first one appeared in the 5′UTR region, and it was identified as c.-39G > A (rs162008). Two mutations appeared in exon 2, c.201G > T (rs35248760) and c.366G > A (rs72557968), and the other two mutations appear in exon 3, c.492G > A (rs1839318) and exon 4 C.671T > C (rs72557975) (Figure 5A).

The *AQP1* gene consists of six exons that span 13.8 Kb. In this case we identified a single nucleotide DNA variant located in exon 1, c.134C > T (rs28362692) and one deletion of five nucleotides within the 3′UTR region that was identified as g.30923685_30923689AGGGG (rs28362739) (Figure 5B).

The human *MOG* gene contains eight exons which span a region of approximately 15.3 Kb. Six variants were found for this gene. The first one appeared again in the 5′UTR region, and it was identified as c.-93T > C (rs9468571). Two variants appeared in exon 1, c.15A > G (rs3130250) and c.47_49TCC (rs71674097), one variant in exon 2, c.306A > G (rs34758289) and, the other variants in exon 3, c.511G > C (rs2857766) and c.520G > A (rs3130253) (Figure 5C).

Sequencing of all exons for the three genes was conducted in 16 MS patients, paying special attention to the distribution of the new variants described above. All the variants described (Figure 5A–C) in NMOsd patients were also observed in the small population of MS patients we analyzed here, except for one variant in the 5′UTR of the *AQP1* gene observed in one patient with NMOsd(–), and in three of the four variants found in the coding region of the *AQP4* gene (rs72557968, rs1839318 and rs72557975), that we observed only in NMOsd(+) patients (Table 2).

## 3. Discussion

An early and accurate diagnosis of NMOsd will lead to more timely and specific treatments that will help the patient to reduce the episodes of recrudescence of the neurological alterations and the accumulation of irreversible neurological damage. The current diagnosis of NMOsd has been stratified according to the detection of anti-AQP4 antibodies in the patient’s serum [5]. Thus, in seropositive patients (NMOsd(+)), the presence of a single typical clinical event together with the exclusion of other diagnostic alternatives is a sufficient condition to establish the diagnosis. On the contrary, in seronegative patients for anti-AQP4 IgG (NMOsd(–)), it is necessary to wait for the appearance of a second clinical episode, in a different location of the CNS, in order to establish the diagnosis with certainty, with the consequent risk of greater accumulation of disabling neurological deficits. Hence, the great importance of being able to accurately identify the presence of anti-AQP4 antibodies, rather than performing a clinical and radiological differential diagnosis of NMOsd patients.

Here we present some clarifications regarding the specificity and efficiency of two of the most widely used clinical assays to detect the presence of anti-AQP4 IgG in NMOsd patients, and explore the usefulness of these antibodies for monitoring the progression of the disease. The analysis of the serum from all patients included in our study started always by determining first the presence or absence of antibodies against AQP4. Our results indicated that these antibodies were only present in patients belonging to group 1, clinically diagnosed as NMOsd (Table 1). The values in the literature indicated that roughly around 70% of NMOsd patients are NMOsd(+), and in our population of patients the number was 67% (12/18) of patients being NMOsd(+), pretty well fitting with the published numbers [1,2,3,4]. Analogous results were obtained for both CBA detection systems used in this study. A discrepancy was only obtained in one sample, which might somehow indicate a higher sensitivity of the commercial CBA kit used versus our own method [23] for detection of anti-AQP4 IgG antibodies. Nevertheless, the precise reason underlying this sensitivity difference between the two CBA assays is not well understood yet. In fact as indicated by previous authors [25], the use of live cells freshly transfected with human M23-AQP4, as was done in our protocol [23], would have a higher accuracy compared to fixed-CBA systems, but we still missed one NMOsd(+) patient that was found positive with the commercial kit.

On the other hand, the ELISA results revealed the lower accuracy of this assay compared to the results obtained with either of the two CBA systems used here. ELISA is a very popular assay used in clinical practice for determination of anti-AQP4 antibodies, which can be easily performed in comparison with CBA. However, and in agreement with previous reports [25,26], our results confirmed that it is a much less sensitive and accurate system. Accordingly, we would indicate that if the anti-AQP4 IgG determination by ELISA fails, especially in patients with clinical and radiological manifestations of NMOsd, re-evaluation of samples with a CBA protocol would be highly recommended. However, the ELISA diagnostic systems offer the advantage of being more quantitative than CBA protocols. Titers of anti-AQP4 IgG could be easily detected by this method, as opposed to the less quantitative CBA systems. Here, since a possible correlation between the levels of these antibodies and the activity of the disease was postulated [8], we decided to follow, along a period of one year, the levels in serum of anti-AQP4 IgG in patients diagnosed as positive for the NMO antibodies. Discouraging results were obtained since no particular increase or decrease or constant values were observed among the 9 patients included in this analysis, suggesting therefore that progression of this disease is independent of the titers of anti-AQP4 antibodies and thus precludes its use as a predictive indicator for future crisis or symptoms during disease evolution. Reinforcing this idea, a relapse occurred in one of the patients monitored during the analysis. For that specific patient, shown in Figure 4 with an asterisk, anti-AQP4 antibodies were always in the low range, even while going through a crisis, therefore indicating that an increase in the titers of anti-AQP4 IgG is not necessary for recrudescence of the disease. However, it can also be seen in the figure that levels of antibodies in that particular patient were clearly higher 3 months after the clinical episode. But no further conclusions can be safely drawn regarding this issue given the small number of patients included in our study.

Apart from the anti-AQP4 antibodies, to find another possible biomarker of NMOsd would be highly desirable. It would improve the diagnosis of this pathology and avoid the need to wait for the appearance of a second outbreak to diagnose this process, in cases of NMOsd(–) patients. The presence in serum of antibodies against AQP1 and MOG, has been associated with the development and diagnosis of NMOsd since some years ago. Thus, the suggestion that the serum determination of anti-AQP1 and anti-MOG antibodies could be another important element in the diagnostic protocol of patients, especially in that group of patients with a high clinical suspicion and without anti-AQP4 antibodies, emerges as an appealing idea to test. Here we present our effort to understand the putative biomarker character for these two proteins, and the possible role of anti-AQP1 and anti-MOG antibodies in the pathophysiology of this complex pathology.

An analysis of the patients’ serum to detect anti-AQP1 and anti-MOG antibodies was also done using both ELISA and CBA. For the detection of anti-MOG IgG, both systems were commercially available from AnaSpec and Euroimmune as indicated in the materials section whereas, for the detection of anti-AQP1 IgG both assays were developed in the laboratory, improving upon methods previously described [24]. Specifically, for anti-AQP1 IgG ELISA determinations instead of using a protein homogenate from cells expressing hAQP1 as before [24], we used human full-length AQP1 recombinant protein, commercially available, to have a pure/high concentration of antigen attached to the bottom plate of the ELISA system. Overall, the results showed that neither of these two antibodies were found in our patients, except for one patient diagnosed with RMy, who turned out to be positive for anti-MOG IgG (Table 1). Regarding anti-AQP1 antibody detection, we feel confident with our detection systems and this study has served to reinforce previous studies [24,27] in which was already confirmed the absence of anti-AQP1 IgG in the serum of any NMOsd patients, anti-AQP4 IgG (+) or (–). For the absence of anti-MOG antibodies, on the other hand, we are still open to explanations before definitively concluding that neither of these antibodies should be considered as new biomarkers for NMOsd. Different isoforms or lengths of the MOG protein used as antigen detection, or use of specific secondary anti human IgG may account in some cases for negative results.

Looking for a genetic association between the etiology of NMOsd and these three genes, our incipient genetic study revealed thirteen DNA variants in patients with NMOsd (Table 2). Specifically, we observed two variants in the *AQP1* gene, one missense alteration located in exon 1 (Ala45Val) and one deletion of five nucleotides within the 3′UTR region. Although Long et al., in 2014. described an abnormal expression of AQP1 associated with NMOsd patients [11], to this day, no genetic variants of the *AQP1* gene have been observed in these patients. The variant we observed in the 3′UTR region has also appeared in MS patients, while the genetic variant in exon 1 has only appeared in heterozygosis in one NMOsd(–) patient, leading us to suspect that these changes are not associated with NMOsd when they are compared with MS.

A similar conclusion must be drawn from the six DNA variants observed in the *MOG* gene of NMOsd patients, since all of them appeared in MS patients as well. The first one located within the 5′UTR region (C.-93T > C); two other mutations in exon 1, a silent variant (Ser5Ser) and a deletion variant (Leu22del); another silent variant located in exon 2 (Lys102Lys) and two missense variants in exon 3 (Val171Leu and Val174Ile). A significant association with the missense variation Val171Leu was previously reported in a small sample of 50 Italian MS patients [28] and, subsequently, this result has been confirmed in an independent Italian sample consisting of 878 MS patients and 890 matched controls [29]. In this analysis, they suggested a dominant-protective effect of the Leu171 allele in MS patients. We have obtained a similar result in our sample, since when we compared the frequency of the minor Leu171 allele in NMOsd versus MS patients, this allele was under-represented in MS patients, 0.22 vs. 0.03, (OR = 0.11; 95% CI: 0.13–0.96; *p* = 0.03) also suggesting a dominant-protective effect. In our study this observation should be interpreted with caution, because we do not have sufficient power to detect a risk factor given the small sample sizes.

Regarding the DNA variants in the AQP4 gene, and in contrast with a study of Japanese patients which sequenced exons of the AQP4 gene and did not find any variant in 16 Japanese patients (13 sporadic cases, and 3 familial cases from 2 families) diagnosed with anti-AQP4 IgG positive NMO [20], we have found five DNA variants in Spanish NMOsd patients. One mutation within the 5′UTR region (c.-39G>A), two silent variants in exon 2 (Pro67Pro and Gln122Gln) and another in exon 3 (Leu164Leu) and, one missense variant in exon 4 (Met224Thr). Furthermore, three of these mutations, Gln122Gln, Leu164Leu, and Met224Thr, have only been observed in NMOsd(+) patients, and never detected in NMOsd(–) or MS patients. Although, the principal limitation of our study is the low number of patients, we think that Gln122Gln and Leu164Leu are not associated with NMOsd(+), because these DNA variants have a similar allelic frequency to that observed in the European population reflected in the Genome Aggregation Database (gnomAD); although it would be interesting to investigate the Met224Thr variant in a larger population of NMOsd(+) patients. The frequency of the Thr224 allele in several European databases searched is 0.001, while the frequency in our NMOsd(+) patients was higher (0.041). Furthermore, Sorani et al., showed that the Met224Thr variant with other three coding variants in the AQP4 gene were associated with reduced water permeability of the cell surface AQP4 protein [30]. Characterization of the effects of these and other AQP4 variants could lead to a better understanding of the function of these important proteins and of their clinical implications. By the moment, these results should be viewed with caution, because our study does not have sufficient power to detect a risk factor due the small sample size.

## 4. Materials and Methods

### 4.1. Subjects and Serum Collection

The study included 119 subjects (81 female) classified into 7 groups based on their medical diagnosis (Table 1). Group 1 was comprised of 18 patients with NMOsd according to the current diagnostic criteria [6]. Group 2 consisted of 48 patients with MS according to the 2010-revised McDonald criteria [31]. In relation to the MS course, we further divided this group into 37 patients with remitting-relapsing MS, 7 with primary progressive MS and 4 with the secondary progressive form. Group 3 was composed of 14 patients with optic neuritis. Twenty-two patients with myelitis comprised Group 4. With respect to the length of the spinal cord lesion, we distinguished between patients whose lesions extended more than 3 vertebral segments (longitudinally extensive myelitis) and those with spinal plaques extended up to 3 vertebral segments (5 and 12 patients, respectively). Furthermore, we categorized the patients of the latter two groups depending whether they suffered an isolated episode (17 patients) or several relapses (5 patients). Group 5 consisted of 4 patients with brainstem clinically isolated syndromes and subjects with other neurological disorders formed Group 6. These last two groups are described in Table 1. Besides the MRI (magnetic resonance imaging) scans and the cerebrospinal fluid analysis, a peripheral blood analysis that included blood count, biochemistry, erythrocyte sedimentation rate, vitamins, thyroid hormones, long chain fatty acids, angiotensin converting enzyme, immunological and serological evaluations were conducted in all patients in order to exclude alternative etiologies to their final diagnosis. Finally, group 7 was composed of 8 healthy controls. After blood was drawn, it was centrifuged at 2500 rpm (15 min, 4 °C) and the serum kept at −80 °C until its use for antibodies detection and DNA was isolated from leukocytes.

The patients (Groups 1–6) and the healthy controls (Group 7) were recruited by the Neurology Department at the Virgen of Rocío University Hospital (HUVR), Virgen Macarena University Hospital, Virgen of Valme University Hospital and the IBiS, respectively. For all participants, written consent was obtained before their inclusion in the study, and demographic and clinical characteristics were recorded, including gender, age at inclusion in the study and clinical diagnosis (Table 1). The project (Ref: PI16/01249) counted with the approval of The Ethics Committee of The University Hospital Virgen del Rocío (HUVR), with the registration number 13/2016 (33160060 PV01) received on 21/12/2016.

### 4.2. Immunofluorescence Assay

In order to analyze the presence of anti-AQP4 IgG, anti-MOG IgG, and anti-AQP1 IgG and compare different techniques for this analysis, two different immunofluorescence assays were performed. For detection of anti-AQP4 IgG and anti-AQP1 IgG we conducted an immunoassay developed in our laboratory. For anti-MOG IgG and anti-AQP4 IgG, an Euroimmun^®^ Assay (Lübeck, Germany) was performed.

#### 4.2.1. Immunoassay Developed in Our Laboratory

Based on a method described previously [23]. Briefly, 24 h before starting the immunoassay, HEK293T-plated cells at about 80% of confluence were transfected with either AQP1-EGFP (described in [24]) or AQP4-EGFP [23] constructs. The cells were then fixed with paraformaldehyde 4% (5 min), washed with PBS for 5 min and then incubated (1 h) in FCS 10% with 1 mg/mL BSA in PBS for the blocking step. Afterward, incubation with the patient’s serum (1:10 dilution for detection of anti-AQP1 and 1:50 dilution for detection of anti-AQP4, 1 h at room temperature) or with an anti-Aquaporin 1 antibody (ab 117970, ABCAM, Cambridge, UK) (1:500 dilution) raised against a full length recombinant human Aquaporin 1 produced in HEK293T cells, was followed by three washes with PBS and then 30 min of incubation with Alexa Fluor 568 goat anti-human secondary antibody (Invitrogen, Carlsbad, CA, USA). Finally, the cells were fixed (1 min) after the secondary antibody incubation, with a mixture of 95% ethanol and 5% acetic acid. Nuclei were stained with 41, 61-diamidino-2-phenylindole (DAPI, 1:1000). The cells were mounted and photographed with an Olympus BX61 microscope equipped with an Olympus DP73 camera. Acquired images were analyzed using NIH ImageJ software (NIH, Bethesda, MD, USA).

#### 4.2.2. Euroimmun^®^ Assay

The screening test for detection of antibodies against AQP4 or MOG-IgG was purchased from Euroimmun (Cat N° FA-1128-1005-1, Lübeck, Germany) and performed as described by the manufacturer. The cells were photographed with an Olympus BX61 microscope equipped with an Olympus DP73 camera. Acquired images were analyzed using NIH ImageJ software (NIH, Bethesda, MD, USA).

### 4.3. ELISA for Anti-AQP4, Anti-MOG, and Anti-AQP1 Antibodies

#### 4.3.1. ELISA for Anti-AQP4 or Anti-MOG IgG

Two different commercial ELISAs were conducted by following the procedure described by the manufacturer. For anti-AQP4 IgG we performed the AQP4 Autoantibody ELISA Version 2 kit from ©BioVendor (Cat N° RAQP4/96/2R; Brno, Czech Republic) and for anti-MOG IgG, the SensoLyte^®^ anti-Human MOG (1-125) Human IgG Specific Quantitative ELISA Kit from ©AnaSpec (Cat Nº AS-55153-H; Fremont, CA, USA).

#### 4.3.2. ELISA for Anti-AQP1

In order to quantify the levels of anti-AQP1 IgG and because there is no commercially-available ELISA for anti-AQP1 antibodies, we improved on the previous assay described in [24]. Briefly, AQP1 (Human) Recombinant Protein (PO1) (Cat N° H00000358-PO1) was purchased from Abnova (Taipei, Taiwan). Proteins were diluted in 0.01 M of buffer carbonate and 10 ng of protein per well or a 1:2 serial dilution for the standard were loaded into a 96 well plate for ELISA (Microwell MaxiSorp, Nunc, Waltham, MA, USA). Subsequently, the plate was covered with a plastic film and placed overnight at 4 °C. The next day the solution was removed and the plate was washed three times by filling the wells with 200 µL PBS1X + 0.05% Tween and once with PBS1X.

Blocking: To block the remaining protein-binding sites in the coated wells, 200 µL of SuperBlock Blocking Buffer (ThermoScientific, Vantaa, Finland) was added per well and incubated at room temperature for 1 h, keeping the plate covered with a plastic film. Then, the blocking solution was removed and the plate was washed three times by filling the wells again with 200 µL PBS1X + 0.05% Tween and once with PBS1X. Two primary antibodies, 100 µL per well, were used; a commercial anti-AQP1 antibody (ab15080, ABCAM) diluted 1:10,000 in PBS with 2% BSA, that serves as a control to establish the assay conditions, and the patients’ undiluted serum. The incubation was allowed to proceed overnight at 4 °C and the next day plates were washed as indicated for removing the blocking solution mentioned above. Then, incubation with the secondary antibodies for 1 h at room temperature was carried out. Horseradish peroxidase conjugated goat anti-rabbit IgG antibody diluted (1:5000) in PBS with 2% BSA for the AQP1 commercial antibody, and horseradish peroxidase conjugated chicken anti-human IgG antibody for the patient serum antibodies were used. Washing the plates at the end was again carried out as before. For signal detection 100 µL of 3,31,5,51-Tetramethylbenzidine (TMB) TMBOne solution (Promega, Madison, WI, USA) was added and incubated at room temperature for 15 min to allow for the enzymatic reaction and development of the coloured substrate. Then, 100 µL of HCl 1N was added per well to stop the reaction and absorbance at 450 nm was measured in a plate reader system (Multiskan Spectrum-Thermo, Vantaa, Finland).

### 4.4. Analysis of AQP4, AQP1 and MOG Genes

We obtained peripheral blood from all patients to isolate germline DNA from leukocytes. DNA extraction was performed according to the standard procedures using QiAamp DNA minikit in a QIAcube Robotic Workstation (Quiagen, Hilden, Germany).

The DNA sequence used to conduct this study corresponds to the genomic sequence of AQP4 (18:26852043-26865771; ENST00000383168), AQP1 (7:30911694-30925517; ENST00000311813), and MOG (6:29657002-29672372; ENST00000376894) genes. The genomic sequence containing these genes was identified using the BLAST tool at Ensembl Genome Bioinformatics server (http://www.ensembl.org). An automated DNA sequencing method was employed to scan the entire coding region of the AQP4, AQP1 and MOG genes in 18 NMOsd (12 seropositive and 6 seronegative for anti-AQP4 IgG) and 12 MS patients. Overlapping PCRs covering the coding sequence of the three genes were designed. PCR products were purified and bi-directionally sequenced using the corresponding primers (Supplementary Table S1). Sequencing reactions were performed using the BigDye Terminator v3.1 Cycle Sequencing Kit (Applied Biosystems, Foster, CA, USA), according to the manufacturer’s instructions, and analyzed using AB3500 Genetic Analyzer and Sequencing Analysis v5.4 software (Applied Biosystems, Foster, CA, USA). Information concerning any variation identified was compared with the UCSC Genome Bioinformatics and also with Genbank Database at the National Center for Biotechnology Information (http://www.ncbi.nlm.nih.gov/).

### 4.5. Statistical Analysis

Data are presented as mean ± standard error of the mean, and analyzed using the Statistical Package for Social Sciences (SPSS Inc., Chicago, IL, USA), version 19.0. Data with a non-normal distribution were analyzed using analysis of variance (ANOVA) for non-parametric data with the Kruskal–Wallis H test.

## 5. Conclusions

Only the presence of antibodies against AQP4, but not anti-AQP1 or anti-MOG IgG, was detected in the serum of the Spanish NMOsd patients we tested here. Based on our results, we consider it very unlikely that these two antibodies, anti-AQP1 and anti-MOG IgG, can serve as new biomarkers for NMOsd in the population studied. As detection system for serum antibodies, our results again confirmed that CBA is the more accurate and sensitive method for detecting anti-AQP4 IgG. ELISA screening was especially helpful when patients were strongly positive for anti-AQP4 antibodies, but would always need confirmation with the CBA system for a definitive diagnosis. Furthermore, our genetic analysis indicates that there are several DNA variations in the *AQP1*, *AQP4*, and *MOG* genes in patients with NMOsd, which are neither necessary nor sufficient for developing this syndrome. However, our data also supports a comprehensive analysis of the *AQP4* gene in a large NMOsd independent population. Prospective studies should include the analysis of the expression of other related candidate genes, and ultimately, the comparison of the gene expression profile using microarray technology.

## Figures and Tables

**Table 1:** Demographic and clinical variables of 119 patients.

Diagnosis	Number of Patients	Gender (Female/Male)	Mean Age at Inclusion ± SD	AQP4			MOG		AQP1	
				CBA	EURO INMUN	ELISA	EURO INMUN	ELISA	BCA	ELISA
1. NMOsd	18	15/3	56 ± 13	11	12	7	-	-	-	-
2. MS	48	32/16	44 ± 12	-	-	-	-	-	-	-
2.1. RRMS	37	28/9	40 ± 10	-	-	-	-	-	-	-
2.2. PPMS	7	2/5	62 ± 12	-	-	-	-	-	-	-
2.3. SPMS	4	2/2	47 ± 11	-	-	-	-	-	-	-
3. Idiopathic	14	9/6	47 ± 10	-	-	-	-	-	-	-
3.1. Isolated episode	3	3/0	34 ± 18	-	-	-	-	-	-	-
3.2. Recurrent idiopathic ON	11	6/5	49 ± 12	-	-	-	-	-	-	-
4. Idiopathic myelitis	22	14/8	49 ± 13	-	-	-	-	-	-	-
4.1. Isolated episode	17	9/8	51 ± 14	-	-	-	-	-	-	-
4.1.1. < 3 vertebral segments	5	2/3	40 ± 5	-	-	-	-	-	-	-
4.1.2. > 3 vertebral segments	12	7/5	55 ± 14	-	-	-	-	-	-	-
4.2. Recurrent idiopathic myelitis	5	5/0	42 ± 9	-	-	-	1	-	-	-
5. Brainstem clinically isolated syndrome	4	3/1	43 ± 19	-	-	-	-	-	-	-
6. Other pathologies	5	3/2	42 ± 16	-	-	-	-	-	-	-
6.1. Glaucoma with visual défic	8	1/0	52 ± 10	-	-	-	-	-	-	-
6.2. Spinal tumor	1	0/1	39 ± 0	-	-	-	-	-	-	-
6.3. Behcet disease	1	1/0	36 ± 0	-	-	-	-	-	-	-
6.4. Leber optic neuropathy	1	1/0	21 ± 0	-	-	-	-	-	-	-
6.5. Ischemic optic neuropathy	1	0/1	62 ± 0	-	-	-	-	-	-	-
7. Healthy controls	8	5/3	36 ± 11	-	-	-	-	-	-	-

AQP: Aquaporin; MOG: oligodendrocyte myelin glycoprotein; SD: standard deviation; CBA: cell-based assays; NMOsd: neuromyelitis optica syndrome disorder; ON: optic neuritis; MS: multiple sclerosis; RRMS: remitting relapsing multiple sclerosis; SPMS: secondary progressive multiple sclerosis; PPMS: primary progressive multiple sclerosis. -: no detected.

**Table 2:** Genetic variants in AQP1, AQP4 and MOG genes observed in NMODS(+) (AQP4-Ab-positive), NMOsd(–) (AQP4-Ab-negative) and Multiple sclerosis (MS) patients.

GENE	SNP	Change	Amino Acid Codon	Amino Acid Change	NMOSD(+) *N* = 12	NMOSD(–) *N* = 6	MS *N* = 16
AQP4	rs162008	c.-39G > A	-	-	5	0	7
	rs35248760	c.201G > T	[CCG] > [CCT]	Pro67Pro	4	4	5
	rs72557968	c.366G > A	[CAG] > [CAA]	Gln122Gln	1	0	0
	rs1839318	c.492G > A	[TTG] > [TTA]	Leu164Leu	1	0	0
	rs72557975	c.671T > C	[ATG] > [ACG]	Met224Thr	1	0	0
AQP1	rs28362692	c.134C > T	[GCG] > [GTG]	Ala45Val	0	1	0
	rs28362739	dupA(G)4	-	-	4	1	1
MOG	rs9468571	c.-93T > C	-	-	1	0	2
	rs3130250	c.15A > G	[TCA] > [TCG]	Ser5Ser	2	0	4
	rs71674097	c.47_49TCC	[CTCC] > [CAA]	Leu22del	1	1	3
	rs34758289	c.306A > G	[AAA] > [AAG]	Lys102Lys	1	0	1
	rs2857766	c.511G > C	[GTT] > [CTT]	Val171Leu	5	3	1
	rs3130253	c.520G > A	[GTC] > [ATC]	Val174Ile	2	0	2

## Supplementary Materials

Supplementary materials can be found at https://www.mdpi.com/1422-0067/20/22/5810/s1.

## Abbreviations

NMO	Neuromyelitis optica
NMOsd	NMO spectrum disorder
NMOsd(+)	NMOsd patients that present antibodies against AQP4
NMOsd(–)	NMOsd patients that do not present antibodies against AQP4
CBA	Cell-based assays
ELISA	Enzyme-Linked ImmunoSorbent Assay
AQP4	Aquaporin-4
AQP1	Aquaporin-1
MOG	Myelin oligodendrocyte glycoprotein
