# Supplementary material for: Predictive Value of Serum Antibodies and Point Mutations of AQP4, AQP1 and MOG in A Cohort of Spanish Patients with Neuromyelitis Optica Spectrum Disorders
Source: Int J Mol Sci. 2019 Nov 19;20(22):5810. doi: 10.3390/ijms20225810 (PMC6887710; doi:10.3390/ijms20225810)
Supplement: Supplementary file 1 [file ijms-20-05810-s001.pdf]

GENE	EXON	Primers Forward/Reverse
**AQP1**	1	5’ TATAAATAGGCCCAGCCCAG 3’<br>5’ CCAGTGCCTTTCAGCATCA 3’
	2-4	5’ GTGCTCCTGACCATCACCTT 3’<br>5’ ACTAGCAGGTGGGTCCCTTT 3’
	2	5’ GGGCTGGAGTTTCATTAACACAG 3’<br>5’ CAGAGCCTCCAGAACAGGAAG3’
	3	5’ CTCACTCTCTCTTCACCTATGAC 3’<br>5’ CAGAGCCTCCAGAACAGGAAG3’
	4	5’ CTGTGGGGTAACCTAGGGAAC 3’<br>5’ ACTAGCAGGTGGGTCCCTTT 3’
**AQP4**	1	5’ GTGATCAGGTACAGAAAAGCTACTTC 3’<br>5’ CTGAAACATATGGAGGATTTGGC 3’
	2	5’ CAGAAGCATTCTTTCTTGGTGTGC 3’<br>5’ CAGAAGCATTCTTTCTTGGTGTGC 3’
	3-4	5’ CTCAGTTGAAGGTGGGGTAGAAG 3’<br>5’ GGTTGGAGAAATGCAAGAGAAAGTAG 3’
	5	5’ GGATCTCTGATGAGGCTTTGG 3’<br>5’ GACATGAAACAACAAACCTGCAC 3’
**MOG**	1	5’CCTCTGCTCCATTGCTCTG3’<br>5’GGAAGGGAGGCATGTCAGTA3’
	2	5’ TCCTCCCTGGCTCTAGAATG 3’<br>5’ CCCAGGACTGAGATGTTTGG 3’
	3	5’ GGGACCAATTCTGTGTCACC 3’<br>5’ TGAACCCAGAAGTCACTCACA 3’
	4-5	5’ CAGGCTGCAGAGAAATAGCC 3’<br>5’ GGAAGGGACTCAACCAGGAG 3’
	6	5’ CTCGGACTCCCAGAGTGTTG 3’<br>5’ CCACTGCACCTGCTAGTCTTC 3’
	7	5’ GTGCCCTGCTGGAAGATAAC 3’<br>5’ TTGCTTCTGTCTCCCTTTCC 3’
	8	5’ GGAGACAAGATGACCCCAAG 3’<br>5’ AGATTCCTGGCCTCCAGTTC 3’

Supplementary Table 1. Primer pairs for PCR amplification and sequencing reaction of exons in *AQP1*, *AQP4* and *MOG* genes
